# Adopting a user-centred design approach for the development of on-device technology to prevent the viewing of child sexual abuse material: app design insights and principles from the development of ‘Salus’

**DOI:** 10.3389/fpsyt.2026.1595063

**Published:** 2026-05-12

**Authors:** Deanna Davy, Suzanne Beaufoy, Stefan Bogaerts, Minne De Boeck, Elien De Caluwé, Erifili Efthymiadou, Rhea Joy, Lea Kamitz, Manon Kleijn, Julia Nentzl, Lee Smith, Larissa Van Puyvelde, Sarah Wefers, Samantha Lundrigan

**Affiliations:** 1 Anglia Ruskin University, Chelmsford, United Kingdom; 2 StopSo UK, Rochester, United Kingdom; 3 Tilburg University, Tilburg, Netherlands; 4 Antwerp University Hospital, Antwerp, Antwerp, Belgium; 5 Lucy Faithfull Foundation, Stoke Prior, United Kingdom; 6 HM Prison and Probation Service, London, United Kingdom; 7 Charité – Universitätsmedizin Berlin’s Institute of Sexology and Sexual Medicine, Berlin, Germany

**Keywords:** child sexual abuse material, offender management, prevention, technology, user-centred design

## Abstract

**Introduction:**

The volume of Child Sexual Abuse Material (CSAM) available online and the global demand for it has reached unprecedented levels. Increasing numbers of individuals concerned about their online behaviour are contacting therapeutic providers for help and support outside of the criminal justice system. Previous research asking individuals what would help them to stop viewing CSAM suggests that the availability of a technological solution to voluntarily self-manage access to CSAM could be an effective tool.

**Aim:**

To explore the findings from the user-centered design (UCD) of the ‘Salus’ prototype - a technological prevention tool to support effective self-management of individuals at risk of committing a first or further CSAM offence(s).

**Materials and methods:**

In this two-year, European Commission funded project we conducted research in four European countries: Belgium, Germany, the Netherlands, and the United Kingdom (UK). For the UCD phase of the project we conducted semi-structured interviews with 31 at-risk individuals in Belgium (n=10), Germany (n=10) and the UK (n=11), to explore the specific needs, design features, deployment methods, and concerns and barriers for the design, functionality and deployment of Salus. Additionally, four focus group discussions (FGDs) were held in Belgium, the Netherlands, and the UK with service providers (primarily therapists and managers) with extensive experience of supporting individuals at risk of committing CSAM offences to explore the same questions at the service level.

**Results:**

In terms of privacy and security, the potential discovery of apps such as Salus, data security and legal consequences of app usage are the main concerns of potential app users. There was consensus on the value of blocking CSAM, but opinions on the inclusion of an optional adult sexual content (pornography) filter in Salus design were not unanimous. Users should be able to switch a pornography filter on and off at their convenience. Blocking notifications should be quiet and subtle. Interactivity features are welcomed by potential users – these may include a diary function; a personal CSAM statistics page; a resources section; and a function to allow users to provide feedback to the app developers. Such features should be optional for users in order to prevent any unintended consequences of app usage. Finally, app deployment must be safe and secure.

**Conclusion:**

Based on these findings, we propose seven evidence-based design principles for user-centered harm-reduction technology: privacy-by-default architecture; discretion through design ambiguity; adaptive notification systems; optional interactivity with user control; trusted-channel deployment; progressive trust building; and fail-safe harm prevention. These principles provide a framework for app developers and researchers working on similar technologies to develop interventions that reduce harmful behaviours.

## Introduction

The distribution and viewing of child sexual abuse material (CSAM) continues to rise, with the Internet Watch Foundation (IWF) reporting in 2023 that the organization dealt with 392,665 reports in that year that were suspected to contain CSAM (1). Longitudinal studies have shown a trend towards more egregious sexual content over time, with more cases involving explicit sexual conduct (2).

CSAM distribution and viewing is enhanced and facilitated by ever-evolving technology, and the accessibility and anonymity of the internet – especially the dark web, peer-to-peer sharing, end-to-end encrypted platforms/chats, social media, etcetera (3, 4). This growing problem contrasts with the limited capacity of countries to deploy resources to combat CSAM. Traditional solutions, such as law enforcement and legislative measures, are insufficient to tackle CSAM in today’s online world and do not provide a sufficient response to this global phenomenon (4–6).

In the context of rapidly expanding technology, insufficient law enforcement approaches, and the increasing demand for CSAM, there are rising voices advocating that the phenomenon should be advanced to a more offender-oriented preventive approach, along with new technologies for reducing the supply of CSAM. This approach actively prioritizes the prevention of harm through evidence-based interventions for persons at risk of offending, or for those at risk of recidivism (4, 7).

The European Union’s (EU) commitment to prevention through targeting the demand for CSAM is set out in Directive 2011/93/EU, whereby Article 22: Preventive intervention programmes or measures states that, ‘Member States shall take the necessary measures to ensure that persons who fear that they might commit any of the offences [ … ] may have access, where appropriate, to effective intervention programmes or measures designed to evaluate and prevent the risk of such offences being committed’. The benefits of effective and widely adopted prevention programmes for CSAM offending are considerable. A public health approach whereby relevant treatment and services are made widely available, and those who are at risk are encouraged to seek support before an offence has occurred would reduce the demand for CSAM, the victimization and revictimization of children, and the significant costs associated with child sexual abuse (8). However, in an Implementation Assessment of the Directive in 2016, it was concluded that the development of a ‘culture of prevention’ was missing at EU level and that only seven Member States had put provision measures in place to meet this provision (EC Transposition Report, COM (2016). A renewed commitment to perpetrator prevention is laid out in the 2020–2025 EU Strategy to Combat CSA. Initiative 5: Enable Member States to better protect children through prevention states, ‘it would have a strong focus on prevention programmes for offenders and for people who fear that they might offend, as this is the area where Member States struggle the most’ (Initiative 5, COM (2020) 607).

While strongly advocated for, widespread preventative therapy for both non-offenders and offenders alike is difficult to attain. Intervention programmes for individuals convicted of a CSAM offence are a vital component of recidivism prevention; however, while all Member States have some form of intervention programme for convicted offenders, their availability, content, and effectiveness vary widely (9). The availability of effective interventions does not seem to be guaranteed across Member States. Treatment can range from short-term therapy, long-term psychotherapy, cognitive behavioural therapy (CBT), and psychoanalysis through to chemical interventions (see 10, 11). Of the available provision for individuals who have not been convicted of an offence, two of the largest EU providers are Stop It Now! and Kein Täter werden (Don’t Offend). Available in several EU Member States, the Stop It Now! helplines provide anonymous telephone support and advice to individuals struggling with inappropriate thoughts regarding children and concerned about their internet behaviour. Over 200,000 people sought advice or support via the Stop it now! online self-help or confidential helpline in 2022 (12). In Germany, Kein Täter werden, a network of 11 treatment sites, attracts an average of 2,500 users per month to their Troubled Desire website and the Berlin site of Don’t Offend has received over 11,000 enquiries about treatment options since the project began in 2005 (13). The numbers contacting these providers alone give some indication of the demand from individuals for help and support; however, we know that these numbers are just the tip of the iceberg. The ReDirection survey (2021), which canvassed the views of 8,484 CSAM users in the Dark Web found that only 13 per cent had sought help but that 50 per cent reported that they would like to stop viewing CSAM, and 62 per cent had tried to stop viewing CSAM but failed (14). Furthermore, most survey respondents (62%) reported concern that if they were able to desist from viewing CSAM, they would struggle to maintain this behavioural change. When asked what would help them stop, many respondents cited several potentially effective methods, including restricting access to CSAM, as well as therapy or counselling (14). When we consider the emerging evidence for the role of CSAM as a precursor to contact offending (e.g., when viewing CSAM: 52% felt it might lead to sexual acts against a child; 44% thought about seeking direct contact with children and 37% had sought direct contact with children (14), the value in providing individuals with a technological solution to voluntarily self-manage their access to CSAM to reduce child sexual abuse offending becomes even more compelling.

Recent technological initiatives have focused on the detection of CSAM, such as the detection of nudity (15); the further improvement of tools that determine estimation of age (17); and technologies that assign so-called ‘hashes to images’ (16); as well as evidence gathering with suspected CSAM offenders (18, 19). Some of these technologies are built using Artificial Intelligence (AI), such as Machine Learning (ML). This is a form of AI, aimed at building systems that can learn from processed data or use data to perform better (20). This type of technology seems to hold a lot of promise in the context of better-developed CSAM prevention-related advances, as well as technological sophistication among those who (potentially) offend. Therefore, it presents an opportunity to use these techniques in the fight against CSAM, especially in the prevention framework (4, 7). To date, however, a tool has not been developed that uses AI and ML to identify and block CSAM images and videos from users’ personal devices, such as mobile phones, iPads and laptops.

‘Best’ practice in CSAM prevention, including in the design and development technological tools for prevention, has not yet been clearly identified in the literature; however, some scholars have attempted to highlight the way forward in developing technological interventions to reduce harms to children, such as CSAM. For example, Razi et al. (21) argued that taking a human-centered approach to AI and ML is important in the detection of online sexual risks, and the scholars advocate for ‘human-centered machine learning’ (HCML). This is a new field of computer science that draws from the social sciences so that AI and ML can respond to societal needs and learn from human insight and ethical considerations (21). Bursztein et al. (3) have pointed out the changes in the distribution of CSAM over the last 25 years, from File Transfer Protocols (FTP) and email being the earliest distribution tools in the late 1990s to early 2000s, which shifted to chatrooms and messenger in the mid-2000s, then to gaming, SMS and mobile phone to the dark web and URLs around 2015 onwards (see 3). In light of this constant evolution in methods for sharing CSAM, the authors recommend using deep learning technology to automate detection of CSAM in order to tackle it, especially new content. For example, they suggest deep learning as a way to scale the content review process by using two types of automated clustering: scene clustering, which involves grouping reported images/videos to look for the same scenes within them (e.g., backgrounds); and facial clustering by developing existing facial recognition technology to further assist in identifying the same victims and perpetrators across images/videos. The authors also recommend using deep learning to help prioritize the ‘most actionable’ content, and to reduce harm to professionals who have to review CSAM (3).

There have also recently been efforts to prevent CSAM offending through other approaches, such as the use of splash pages and deterrence messages, and to measure the effectiveness of such prevention measures. Many individuals looking for CSAM for the first time do so via surface web-based search engines (22). Having a mechanism, such as a splash page, provide a direct message on the device screen saying that it is not normal, acceptable or legal to search for CSAM may function as a prevention measure (23). The organisation, Thorn, has used prevention messages encouraging people to seek help through resources, including Stop it Now!’s confidential helpline. When Internet users search for CSAM, they see a splash page with information about the illegality of viewing CSAM and resources for people struggling with a sexual preference for minors and children. Stop it Now! in the United States indicated that they received visits to their website from between 200 and 250 people a month as a result of Thorn’s splash pages (24). Thorn further stated that of the individuals who received the deterrence message, more than 13 per cent sought more information about receiving help, which accounted for more than 17,000 people (24). Similar positive results have been identified in the Australian context: Prichard et al., (25) study of the effectiveness of deterrence messages found that three quarters of the control group (who were not directed to a deterrence message) attempted to enter a pornography site, compared with 35 per cent to 47 per cent of the experimental group (who received a deterrence message). Both Thorn and Stop it Now! stress that it is important to include help information in any deterrence message that is also targeting behavioural change. Such behavioural change can start with a deterrence message but should be complemented and effectuated by continued engagement with those seeking help (23).

Taking into account the growing volume of CSAM worldwide, the constant evolution in methods for sharing CSAM, and the importance of working preventively, the Protech^1^ consortium aimed to develop an on-device technology, driven by ML, to prevent the viewing and distribution of CSAM. To this end, the Protech team developed ‘Salus’, which is an on-device app (for use on smart phones, iPads, laptops, and personal computers) that blocks CSAM viewing on users’ devices. The Salus prototype was developed in line with a user-centered design (UCD) approach. Interviews were conducted with individuals at self-reported risk of viewing CSAM, and focus group discussions (FGDs) were conducted with service providers (therapists, managers). Participants were invited to provide their thoughts and concerns regarding four key domains of questioning, which were: (1) Salus privacy and security features; (2) CSAM blocking design and functionality; (3) potential interactivity features for users; (4) and possible deployment methods. Participants were also invited to share their thoughts on any other aspect of Salus design and functionality, as well as their concerns regarding Salus, which had not already been covered in the above-mentioned four domains.

UCD is an evidence-based method for developing interventions and prioritizing the needs of users. It is considered a way to ensure functionality and effectiveness in order to make the app workable in the end users’ lives (26). Therefore, UCD includes participation and involvement of end users and consequently empowers them to actively contribute to the design process. Key features of UCD are a design based on an understanding of users, environments and interactions in context as well as user involvement; hence, a design driven and refined by user-centered evaluation (27). In addition, the approach also includes three core values throughout the development process, with consensus being always a common thread throughout: First, it is important to actively involve end users from the beginning and not just at the end of the process; second, involving end users can be challenging, depending on the target group and inclusion criteria so it is important to define the target user audience as strictly as possible; third, all input given by users during the development process should be included in the design to the extent of what is feasible (28).

The UCD of Salus followed a semi-iterative process. While we aimed to follow good practice in UCD design, we were limited by the project timeline (two years), the immense challenges in developing and refining the Salus app within the timeline for initial prototype design and refinement, and project funding constraints, which meant that more app developers could not be employed in order to speed up the process of app development and refinement. Furthermore, due to time constraints in the collection of data from potential users, we established pre-determined domains for questioning (privacy, blocking, interactivity, deployment) - this limited inductive flexibility, and thus also limited the potential emergence of alternative themes.

This paper presents only the findings from research on the UCD approach to the development of Salus. In this paper we focus on exploring participants’ thoughts on the design, functionality, and deployment of Salus, highlighting generalizable insights into CSAM blocking technology that may be considered by organizations developing similar technological solutions for the reduction of harms. The UCD phase of Salus was the second phase of the two-year Protech project. The first project phase involved initial prototype design by the app developers. The second phase of the project was the UCD phase of Salus, which involved adult volunteers (at self-reported risk of viewing CSAM) viewing prototype images and being asked a series of questions by their service provider to gather their thoughts on app privacy and security, blocking, interactivity and deployment. Subsequent project phases included: Testing and refinement of the prototype; piloting and evaluation of Salus (an implementation, process and outcome evaluation over nine months); and analysis of results and dissemination of findings through workshops, conference presentations, podcasts, and academic publications.

Beyond exploring specific design considerations for Salus, this paper aims to contribute to the emerging theoretical foundation for harm-reduction technology by distilling empirical insights into formalized design principles. While prior research has emphasized human-centered approaches to detection technologies (21) and the evolution of CSAM distribution methods (3), there remains limited conceptual guidance for developers creating prevention-oriented, user-facing tools. The paper addresses this gap by translating user-centered research into actionable design principles applicable across harm-reduction contexts.

The structure of this paper is organized as follows. Following the Introduction, the second section presents the study method, including the study aim, objectives and research questions; participants; procedure; materials; and analyses. The third section explores the results of the research, focusing on participants’ insights regarding the four domains listed above, as well as other themes identified outside of the four domains. We wrap up each sub-theme with a short section on design implications, highlighting the key design principles that were identified, and the rationale for them. The fourth section provides a discussion of the results against a backdrop of recent literature on design considerations in technological interventions for at-risk populations. The discussion also presents a summary of the seven design principles identified in the study, presenting them as a conceptual framework for app design beyond the specific context of Salus and CSAM prevention. The paper concludes with a brief conclusion of the key points, and a reflection on the way forward in testing the identified principles across different technological interventions and populations in order to validate and enhance the proposed framework.

## Methods

### Aim, objectives, and research questions

The key aim of the study was to explore the findings from the UCD phase of the ‘Salus’ prototype. Salus is essentially a CSAM prevention app that works on individuals’ personal devices, including smart phones, iPads, laptops, and personal computers.

The study objectives included:

- Conduct interviews with individuals (adults) at self-reported risk of viewing CSAM, and FGDs with service providers (therapists, managers and others who provide support to adults at self-reported risk of viewing CSAM) to gather their thoughts on the design, functionality, and deployment of Salus.
- Identify areas of consensus in the interviews and FGDs on the design, functionality, and deployment of Salus.
- Identify key design insights that may be transferred to the design and deployment of similar apps that aim to reduce harms.

The research questions were:

- What are participants’ thoughts and concerns regarding the design, functionality, and deployment of Salus, particularly across the four key domains of interest to the app developer (1) privacy and security, 2) CSAM blocking, 3) interactivity features, and 4) deployment?
- What are the areas of consensus in the interviews and FGDs regarding the design, functionality and deployment of Salus, which should be shared with the app developer during the app design stage and embedded into the protype?
- What are the potential generalizable insights that could be distilled into formalized design rules?

### Design

The multi-phase EU Commission-funded Protech project was carried out in four European countries in 2023-2025: Belgium (University Forensic Center/Stop it Now! Flanders (UFC-UZA), Germany (Institute of Sexology and Sexual Medicine, Charité) (CUB), the Netherlands (Tilburg University (TIL), Offlimits (OL)), and the United Kingdom (Anglia Ruskin University (ARU), Lucy Faithfull Foundation (LFF)). Across the four countries, service providers collaborated with academic partners to draft ethics applications, recruit potential study participants, collect data, and contribute to the drafting of written outputs. Ethics approval was granted separately for each site by institutional review boards affiliated with academic partners in the project.

This UCD phase of the study was the second phase within the two-year project. The first phase of the project was initial prototype design. The second phase was the UCD design of Salus – inviting potential app users and service providers to share their thoughts and opinions on Salus design, functionality and deployment. The third phase involved further refinement of the app, based on the insights from the UCD phase. For example, the app developers carefully considered interviewees’ and FGD participants’ thoughts on security and privacy features, block notifications, interactivity features, and deployment options, and many of these suggestions were embedded into the refined prototype. The fourth phase involved the piloting and evaluation of the app in 2024–2025 in Belgium, Germany, and the UK (9 months). It is in this fourth phase that the effectiveness of Salus measured through an implementation, process and design evaluation involving adult pilot participants in three country settings. As noted in the Introduction, this paper reports only the results of the UCD phase of the project. Future papers will present the results from the piloting and evaluation of Salus.

The UCD phase of Salus design involved qualitative exploration of participants’ thoughts on the desired features and functionality of Salus via semi-structured interviews with individuals at self-reported risk of viewing CSAM who were, at the time of the study, receiving preventative support from sites involved in the study; and via FGDs with service providers in each site, who were experienced in delivering preventative support to at-risk individuals. Interview participants were invited to share their thoughts and concerns regarding the above-mentioned four domains of questioning regarding the design, functionality, and deployment of Salus. FGD participants were invited to discuss similar areas of questioning.

It should be highlighted that our approach to the UCD of Salus was semi-iterative – participants were invited only once to share their thoughts and opinions on Salus design. This was largely due to project time constraints; however, as noted above, a subsequent phase of the project involved adults at self-reported risk of viewing CSAM piloting Salus, and an implementation, process, and outcome evaluation of the pilot – results from this phase will be shared in future academic papers. A truly user-centered, iterative process would have involved engaging the same individuals in the project all the way through the process of prototype design, initial feedback, piloting, and reporting of pilot experiences. It should also be highlighted that by starting data collection with four domains of questioning, we limited inductive flexibility; however, participants were encouraged to provide their thoughts and concerns on Salus outside of the four domains of questioning.

Individuals at-risk of viewing CSAM were individuals at self-reported risk. These were individuals who had approached one of the service providers for assistance in stopping viewing CSAM after becoming concerned about their thoughts or behaviour. In Belgium and Germany, these individuals were generally engaged in individual and/or group therapeutic support, while in the UK most of the individuals were persons who called the Stop it Now! helpline. The interview sample bias should be highlighted – because participants were recruited through service providers, and were individuals who were actively seeking help to stop their CSAM viewing, their perspectives on Salus design are undoubtedly skewed in favour of the app. A wider sample of interviewees may have provided different results in terms of the design and functionality of Salus, and insights regarding the value of such a tool for preventing CSAM consumption.

### Participants

Participant targets were agreed among the Protech consortium partners at the point of project Protech design. It was agreed that 30–50 individuals at self-reported risk of viewing CSAM would be interviewed for the UCD phase of the study, with this target number providing a sufficient sample size to answer the key research questions, and being a realistic target in terms of the number of clients and/or helpline callers at each site. Targets for the FGDs were also established at project inception, with the project partners agreeing to conduct one FGD in each of the participating countries (Belgium, Germany, the Netherlands, and the UK).

Service providers identified eligible participants from the pool of service users accessing therapeutic, psychoeducational, or helpline support delivered through their sites. Eligibility for participation was determined by the following criteria: 1) aged 18 years or above; 2) at self-reported risk of viewing CSAM; and 3) voluntary participation. Exclusion criteria involved service users presenting with high levels of stress, emotional distress or presenting an immediate suicide risk, which could interfere with their ability to provide informed consent. Service users would, however, be considered eligible for participation and introduced to the study if their condition had stabilized by their next contact with their service provider and their capacity of informed consent restored. Approximately 40 individuals were approached about the study; of this number, 31 individuals who met the inclusion criteria participated in interviews. While interviews were initially envisaged in all four countries, interviews were only conducted in three countries (Belgium, Germany and the UK) as no helpline callers in the Netherlands expressed an interest in participating in the study.

Because of the sensitivities around interviewing un-arrested individuals who are at risk of viewing CSAM, and the nature of the client-service provider set up in some countries (for example, in the UK, clients may only utilise the helpline and not wish to engage in any other support services), the research team did not seek to collect much demographic information from potential interviewees. Only data on the sex of participants, and their country was recorded. All interviewees were male. Ten participants resided in Belgium; 10 in Germany; and 11 in the UK. Demographic information on the participants who joined the subsequent pilot and evaluation were collected, and this demographic data will be presented in a forthcoming paper on the evaluation results.

The research team further identified and invited experienced service providers (managers, therapists, and other professionals directly supporting at-risk individuals) to participate in FGDs conducted by academic partners involved in the study. In total, four FGDs were conducted. One FGD was conducted by TIL in the Netherlands (n = 5 participants); one by UZA-UFC in Belgium (n = 8); and two FGDs were conducted by ARU in the United Kingdom (n = 10). Focus groups were conducted by staff within these organisations with experience conducting mixed-methods research. The reason that two FGDs were conducted in the UK was because ten staff expressed interest in participating in an FGD. ARU researchers determined that it would be more efficient to conduct two smaller FGDs rather than one large FGD with ten participants. One FGD was originally envisaged also in Germany; however, the FGD was not conducted due to staff being busy too providing therapeutic support to clients at the time. Twenty-four relevant professionals were contacted regarding the FGDs in Belgium, the Netherlands and the UK and only one person declined to participate due to their busy schedule. In total, 23 service provider staff participated in the FGDs in 2023. Only information on the focus group participants’ country, and role was collected– this information is presented in **Table 1** below. FGD participants had between two and 20 years of service in their respective organisations.

**Table 1:** FGD participants’ demographic information.

Country of focus group discussion	Number of participants	Role
Belgium FGD	8	Forensic therapist/psychologist
		Forensic therapist/psychologist
		Forensic therapist/psychologist
		Forensic therapist/psychologist
		Forensic therapist/psychologist
		Forensic therapist/psychologist
		Manager
		Forensic psychiatrist
Netherlands FGD	5	Helpline worker
		Forensic psychologist
		Forensic psychologist supervisor
		Manager
		Psychologist
United Kingdom FGD	10	Researcher/practitioner
		Head of clinical services (adults)
		Project worker (practitioner)
		Practitioner
		Helpline manager
		Director
		Practitioner
		Head of psychology
		Child sexual abuse prevention manager
		Project worker (practitioner)

### Procedure

#### Semi-structured interviews with at-risk individuals

Recruitment of at-risk individuals took place over the three-month period June 2023 to August 2023, and data collection was conducted over a four-month period (June to September 2023). The informed consent process and all interviews were carried out by the service providers in each site. Information and informed consent documents (translated for each recruitment site) were handed out to eligible participants and signed either in hardcopy format, electronically via email, or alternatively the informed consent process was conducted and recorded anonymously over the phone (in the case of service users, for example, accessing confidential helpline services), or using voice recorders (at the start of the interview). Upon consent, the interview was scheduled by the service provider at a mutually convenient time and, depending on the recruitment setting and the need for participants to maintain their anonymity, interviews were conducted in person or using remote methods (e.g., phone calls, Zoom or Microsoft Teams calls). When the informed consent process was conducted over the phone, there was also the possibility to progress with the interview immediately after consent was given, as opposed to scheduling the interview at a future time. Each interview took approximately one hour. Participants who completed their interviews were debriefed and, if they wished, provided reimbursement for their time in line with the National Institute for Health and Care Research (NIHR) payments guidance in the form of a voucher of GBP^2^25 (or its equivalent in euro currency).

Focus-group discussions with service providers.

Recruitment of service providers for FGDs took place over the two-month period June to July 2023, and FGD data collection was completed by August 2023. Project managers across recruitment sites introduced the study to selected members of their team, distributing information and informed consent documents (translated as necessary) in hardcopy or electronic format. The FGDs were organized collaboratively by project managers and academic partners and conducted in person or using remote methods (e.g. via Zoom or Microsoft Teams). Each FGD took approximately 90 minutes. At the end of the FGD, participants were debriefed. Contrary to interviews with at-risk individuals, FGD participants were not offered reimbursement for attending an FGD, as their participation involved minimal risk, and the FGD was conducted during normal work hours and formed part of their usual work responsibilities.

### Materials

As the app was only an early prototype at the point of data collection, the research team prepared a series of images of the prototype to show participants what the app would possibly look like (that is, the colour scheme, how the app icon might look on a device etc), and key areas of functionality (such as the CSAM block notification, and false block reporting mechanism). Each participant was shown the images at the start of the interview or FGD, explained what each image meant in terms of Salus design and functionality, and allowed time to ask questions, prior to the interview/FGD commencing.

A similar proforma was used for both groups of participants (interviewees, and FGD participants), exploring their thoughts around the design of Salus across four areas: 1) privacy and security, 2) CSAM blocking, 3) interactivity features, and 4) deployment. These four domains were agreed among the project partners, in consultation with the app developer. At the point of project planning, it was agreed that domains 2–4 were critical in terms of app development – the app developer needed to know what potential users, and service providers thought the CSAM blocking notification should look and sound like, and what content potential users might want blocked (i.e. adult sexual content, in addition to CSAM blocking); whether potential users might want the app to be interactive, and if so, what interactivity features should be considered; and how the app should be safely deployed (that is, whether potential users would feel more safe downloading an app from an app store, or through a secure download link provided by their service provider, or other potential deployment options). Service providers highlighted in the early stages of project design the importance of privacy and security features in all aspects of app design, deployment, and data collection, thus domain 1 was a critical one for discussion during the interviews and FGDs. Beyond these four key domains, it was agreed that interviewers (the service providers at each of the three sites) could ask additional questions regarding participants’ thoughts and concerns regarding the design and deployment of Salus. For that reason, a semi-structured proforma was used rather than a structured proforma. A key limitation of the semi-structured approach, however, was that service providers – who do not have extensive experience in conducting interviews – frequently posed interview questions using different words, and also used different prompt questions. Thus, any reported numerical indicators in this paper (e.g., “11 interviewees reported X”) serve only as illustrative measures of consensus rather than definitive quantitative evidence.

All interviews and FGDs were audio-recorded or extensive notes were taken. Each partner that conducted interviews was responsible for taking a note if any personal identifying information was shared by interviewees, and clipping that section from the audio-recording. Recordings were securely transferred to ARU and then transcribed verbatim by a UK-based transcription and translation company. Interviews and FGDs conducted in a non-English speaking country were translated by the company’s linguists from their original language into English. Each partner was alerted when transcripts were ready for their review. This review time allowed the partners an opportunity to check and remove any identifying information regarding the participants, and check the transcription accuracy, prior to the transcripts being analysed by ARU.

### Analyses

ARU was responsible for analysing all interview and FGD data. Each transcript was analysed using content analysis. Content analysis is ‘a research tool used to determine the presence of certain words, themes, or concepts within some given qualitative data (i.e. text). Using content analysis, researchers can quantify and analyse the presence, meanings, and relationships of such certain words, themes, or concepts’ (29).

The content analysis methodology employed by the researchers was summative content analysis. Summative content analysis is a qualitative research method that ‘involves counting and comparisons, usually of keywords or content, followed by the interpretation of the underlying context’ (30). The process involves coding textual data (in this case, interview and FGD data) to identify key categories (noting that the researchers commenced the analysis with four broad domains of analysis already established), then analysing the occurrence of the categories to draw conclusions of the data.

To analyse the transcripts using content analysis, researchers broke the text down into manageable categories (‘codes’). A table was developed based on the four major domains covered by the interview and FGD questions: 1) privacy, 2) blocking, 3) interactivity, and 4) deployment. Additional codes were included in the content analysis through a more inductive approach, that is, the researchers identified themes, such as concerns regarding Salus use, or potential benefits of Salus, and added these themes/codes to the content analysis table.

All relevant information uncovered within each interview or FGD was manually entered into the corresponding cell within the content analysis table by one researcher. Useful quotes were also captured during this process. Then a second researcher reviewed the transcripts and the table, in order to ensure that no key findings had been missed, and that any ambiguities in the data were discussed and addressed.

A key limitation of this approach was that, as noted in previous paragraphs, service providers posed interview questions in different ways, and asked different prompt questions, meaning that any results reported using numerical indicators in this paper can only serve as illustrative measures of consensus across the interviews, and should not be regarded as quantitative evidence.

### Reporting of results to the app developer

The results of the content analysis were communicated to the Salus design company, SafeToNet, on a rolling basis during the final quarter of 2023, to inform the user-centered design of Salus. Key insights from the interviews and FGDs regarding app privacy security, block notifications, interactive features, and deployment preferences were relayed to SafeToNet in order to enable the app developers to embed core features into the prototype. Most, but not all, of the study participants’ thoughts on the design, functionality and deployment of Salus were included in the design and development of the app. For example, participants’ thoughts on app appearance and basic app functionality were included, as were functions to address participants’ privacy and security concerns; however, some other suggested interactivity features, such as a personal diary, could not be included in the prototype simply due to time and resource constraints. The entire project (app design, UCD phase, piloting, evaluation, and preparation of written outputs) had a set timeline of two years, as well as agreed funding parameters. Thus the project team prioritized ‘must haves’ in Salus design and functionality, whereas ‘nice to haves’ were suspended for a future iteration of the app. ‘Must haves’ included in the prototype were – design features (primarily security and privacy), blocking features (particularly block notification messages and alerts), and false block reporting features, while the ‘nice to haves’ mostly related to interactivity features such as a reflective diary and help resources; the latter were not included in the prototype.

Participants’ suggestions for the design and functionality of Salus were relayed to the app developer by the research team, as, due to the importance of participant anonymity, participants could not speak directly to the app developer themselves. Due to project time constraints, this feedback collection was a one-off activity; participants’ views were not sought again on the app design. However, the app was later piloted and evaluated by the research team, with results from the evaluation of Salus to be disseminated in forthcoming papers.

## Results

As noted in previous sections of this paper, participants were invited to share their thoughts, opinions, and concerns during interviews or FGDs on four main areas of Salus design and deployment: 1) issues related to privacy and security, 2) Salus blocking functionality, 3) potential interactivity within the app, and 4) possible deployment methods, as well as their thoughts and concerns beyond these four key domains. The following sections of the paper present the key findings across these four areas, in particular. A final section briefly explores study participants’ thoughts on the overall potential benefits of Salus for users, and concerns associated with Salus that were not already discussed in the above-mentioned four areas of focus. At the end of each sub-section, we highlight the design principles identified that may provide a design framework for app developers and researchers working on similar technologies to reduce harmful behaviours.

As noted in the Methods section, we conducted semi-structured interviews and FGDs and conducted content analysis of the transcripts. Thus it should be noted that any participant counts presented in this Results section of the paper are indicative of thematic prevalence, rather than statistically representative data.

### Privacy and security

#### Privacy and security concerns

Interview and FGD participants highlighted the need for the Salus prototype to ensure the privacy and security of its users. Participants expressed concerns regarding app users’ privacy and security across three key areas: the potential discovery of app usage by users’ family members and/or friends; data security; and legal consequences of app usage (e.g., background reporting of the app to law enforcement).

With regards to concerns about the potential discovery of the app on the user’s device, this concern was highlighted by 11 interviewees and in three focus groups. The major concern discussed here was that intimate partners (a spouse or partner), children, other family members or friends, or even work colleagues would notice the app on the user’s device through a glance at the device or be alerted to the presence of the app through an app alert (a sound). Some interviewees further highlighted that the app could be identified through other methods, for example, by an IT colleague doing routine work on a user’s device and discovering the Salus app.

Interviewees highlighted that at-risk individuals would find this discovery of the app by family members, friends, or colleagues a very stressful event that would not only uncover usage of Salus but also alert these family members, friends, or colleagues to the individual’s CSAM viewing habits (if someone googled Salus and found the Protech website).

Interviewees also discussed their concerns about data security and data breaches. Two interview participants expressed concern that the app might be targeted by hackers, and users’ personal information used by hackers to blackmail them:

*“Imagine a hacker getting into the software and seeing all of the data, those are blackmailing opportunities.” – Interview participant 25 (United Kingdom).*

A related concern voiced by 11 interviewees and in one FGD, was that users’ personal data might be collected through the app or by their service provider, and this information inadvertently leaked, consequently exposing their CSAM offending history.

Finally, five interviewees and participants in two FGDs expressed concern that Salus users may experience legal consequences if law enforcement was to discover Salus on their device if their device was confiscated as part of an investigation:

*“I think the first reaction is worrying whether it will end up with the police.” – Interview participant 19 (Belgium).*

Some interviewees even expressed concern that the service provider may share the individual’s use of Salus with law enforcement. FGD participants explained that this concern predominantly affects un-arrested individuals who have limited trust in service provider organizations:

*“[ … ] but those who are in the criminal justice system and, for example, perhaps on a [name withheld] programme, they’ll be more used to what we’re saying and what we’re doing. It’ll be the other group I think that will present, eventually, with difficulty.” - FGD participant (United Kingdom).*

Interview and FGD participants suggested a number of Salus privacy and security features that would go some way towards alleviating the above-discussed privacy and security concerns. With regards to reducing the risk of app discovery by family members, friends, or work colleagues, 18 interview participants and participants from three FGDs suggested that the app icon should be discreet and neutral.

*“Keeping it as neutral as possible is the way to go, I’d say.” – Interview participant 14 (Belgium).*

**Figure 1:**
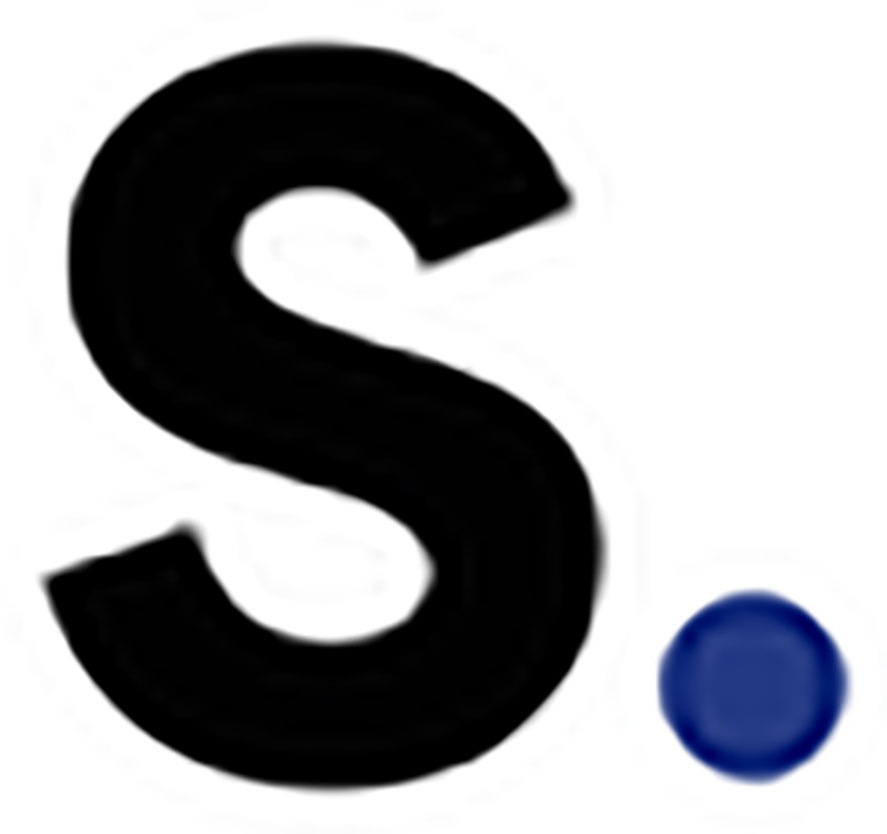
Salus prototype icon.

Some participants (one interview participant and participants from two FGDs) further suggested that the app should not include the full name of ‘Salus’, to avoid family members, friends or colleagues being able to look up what Salus is and discovering, on the Protech website or other internet pages, the purpose of the app. Here, five interviewees and participants from one FGD stated that they were concerned that media coverage about the Protech project may make Salus a well-known name, and thus recommended that the app does not include the full name of Salus and instead, only shows an abbreviation or even an initial, such as ‘S’, see **Figure 1**.

With regards to alleviating concerns regarding data security, interview and FGD participants suggested that Salus should not trace app users or their location (two interviewees; three FGDs); that only minimal and necessary data should be passed on to the server (two interviewees), and that this should be done in an encrypted and anonymized format (four interviewees; two FGDs); that the server should be based in a secure location in a country with strict data protection laws (one interviewee); and that data should not be shared with any third parties (three interviewees; one FGD).

*“I would prefer it if no data were actually sent to the server at all.” – Interview participant 5 (Germany).*

Eight interviewees and participants from four FGDs further highlighted the importance of sharing Salus privacy and security features with users. The suggestion was for a privacy statement or similar document to be developed, translated, and shared with prospective app users that outlines: The data that the app will collect; how the data are stored; and who will be able to view the data. Such a policy should be clear, brief, transparent, and easily accessible:

*“Clear and transparent communication with users from the outset is vital. Users should not have to dig deep to understand what happens with their data.” - FGD participant (Belgium).*

Interview and FGD participants reported that, to alleviate users’ concerns regarding legal consequences of app usage, the app’s privacy statement should emphasize that there are no legal consequences arising from usage:

*“Maybe emphasize the fact that there will not be any jurisdictional consequences.” – Interview participant 16 (Belgium).*

Two interview participants and participants from one FGD further suggested that the privacy statement should clearly explain that the app has not been developed by, or is in any way connected to, the national government, especially law enforcement agencies. Two interviewees and participants from one FGD also noted that the privacy statement should be a positive document, highlighting that the app has been developed to support the user; not to monitor or control them in any way.

In addition to the privacy statement clearly outlining Salus privacy and security features, one interviewee and participants from one FGD suggested that a Frequently Asked Questions (FAQ) document should be made available on the app, as well as disseminated in advance of an individual signing the consent form. This would act as an additional layer of reassurance to potential Salus users who may be concerned about the app’s privacy and security features.

**Figure 2:**
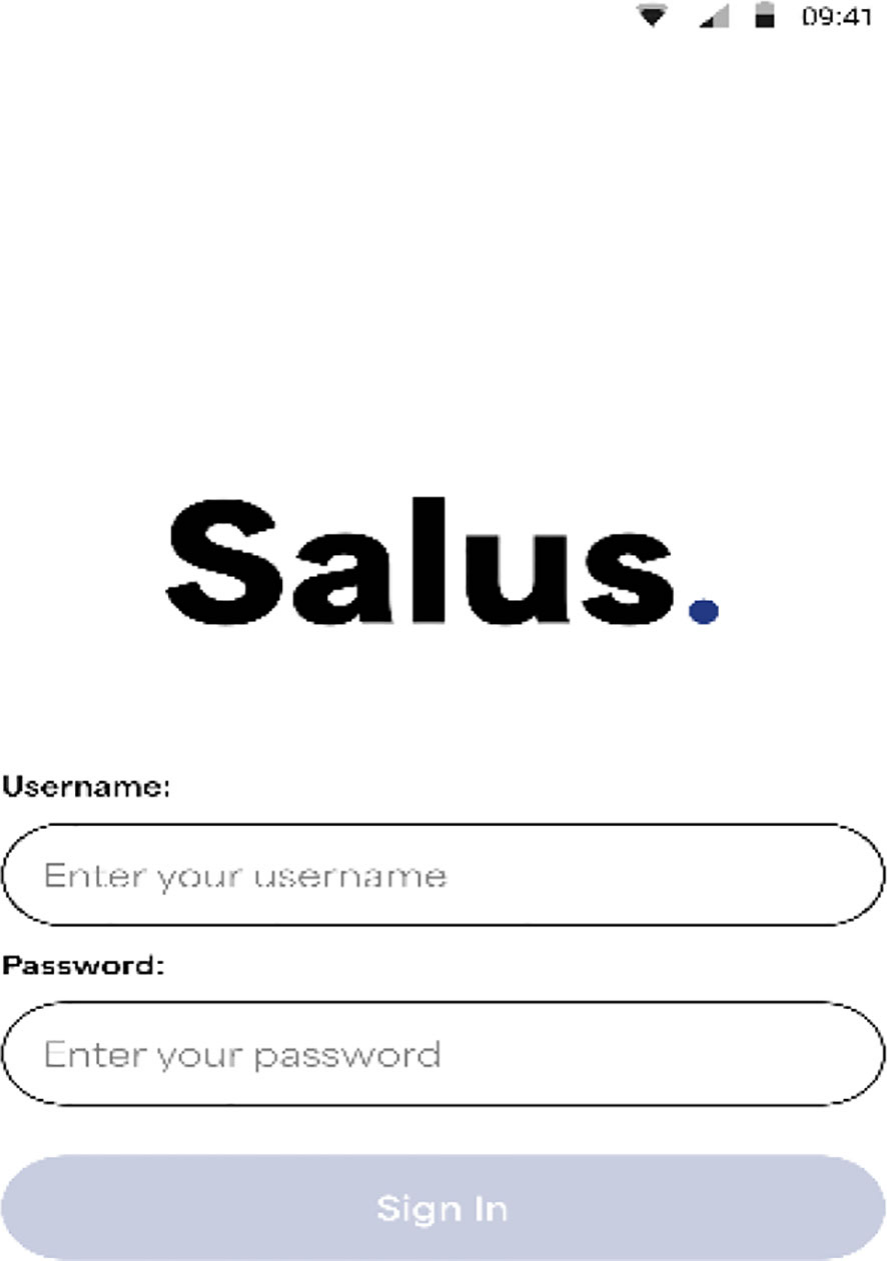
Salus prototype log in page.

Finally, participants generally agreed (10 interviewees, and participants from 3 FGDs) that beyond the initial Salus log in, users should not be required to log in a subsequent time, and that app should just run quietly in the background of the user’s device (unless the user logs out), see **Figure 2**.

These findings highlight several important design principles that should be considered by app developers and researchers designing apps that aim to reduce harms. The first principle is privacy-by-default architecture. Participants expressed significant concerns regarding hacking, blackmailing, and law enforcement discovery of the app (e.g. “I think the first reaction is worrying whether it will end up with the police”). Thus, rather than treating privacy as a feature to be added, technologies must embed privacy protections at the system level to alleviate these concerns. This includes: (1) minimizing data collection to only absolutely essential functions, (2) implementing encryption and anonymization by default rather than option, (3) designing for local operation rather than server-dependent functionality, and (4) creating transparency mechanisms that proactively address user concerns. This principle extends beyond CSAM prevention to any technology serving populations facing potential legal or social consequences from disclosure.

A second design principle is progressive trust building. Participants highlighted the need for up-front information about the app in order to ensure users’ confidence in app security (“clear and transparent communication with users from the outset is vital”). Thus, app developers should design information architecture that builds user confidence through transparency. This should include clear, accessible privacy statements, FAQ sections, and evidence of independence from law enforcement.

A third key design principle is discretion through design ambiguity. The results highlighted that potential app users’ fear of app discovery and subsequent social exposure is a significant concern, and ambiguous app iconography would be beneficial. Thus, it is critical that design solutions protect app users from unintended discovery by family members, friends, colleagues, and others. App designers should create deliberately ambiguous app visual and naming conventions that prevent app discovery, such as neutral iconography, abbreviated naming, and quiet background operation after initial setup.

### Block features

A non-negotiable aspect of Salus design was that the prototype must block CSAM; however, interview and FGD participants were asked about their thoughts on other block options, as well as CSAM block messaging and block tones.

#### Material that should be blocked

Most participants (26 interviewees; participants from four FGDs) suggested that Salus should also block adult sexual content because adult content acts as a pathway for some individuals to view CSAM. Their opinion was that Salus should block adult content as this would reduce the risk that users would subsequently want to view CSAM. FGD participants further explained that many of their clients report a desire to also stop looking at pornography, because of this downward spiral into wanting to view CSAM:

*“We have a large number of clients who also don’t want to be accessing adult pornography.” – FGD participant (United Kingdom).*

**Figure 3:**
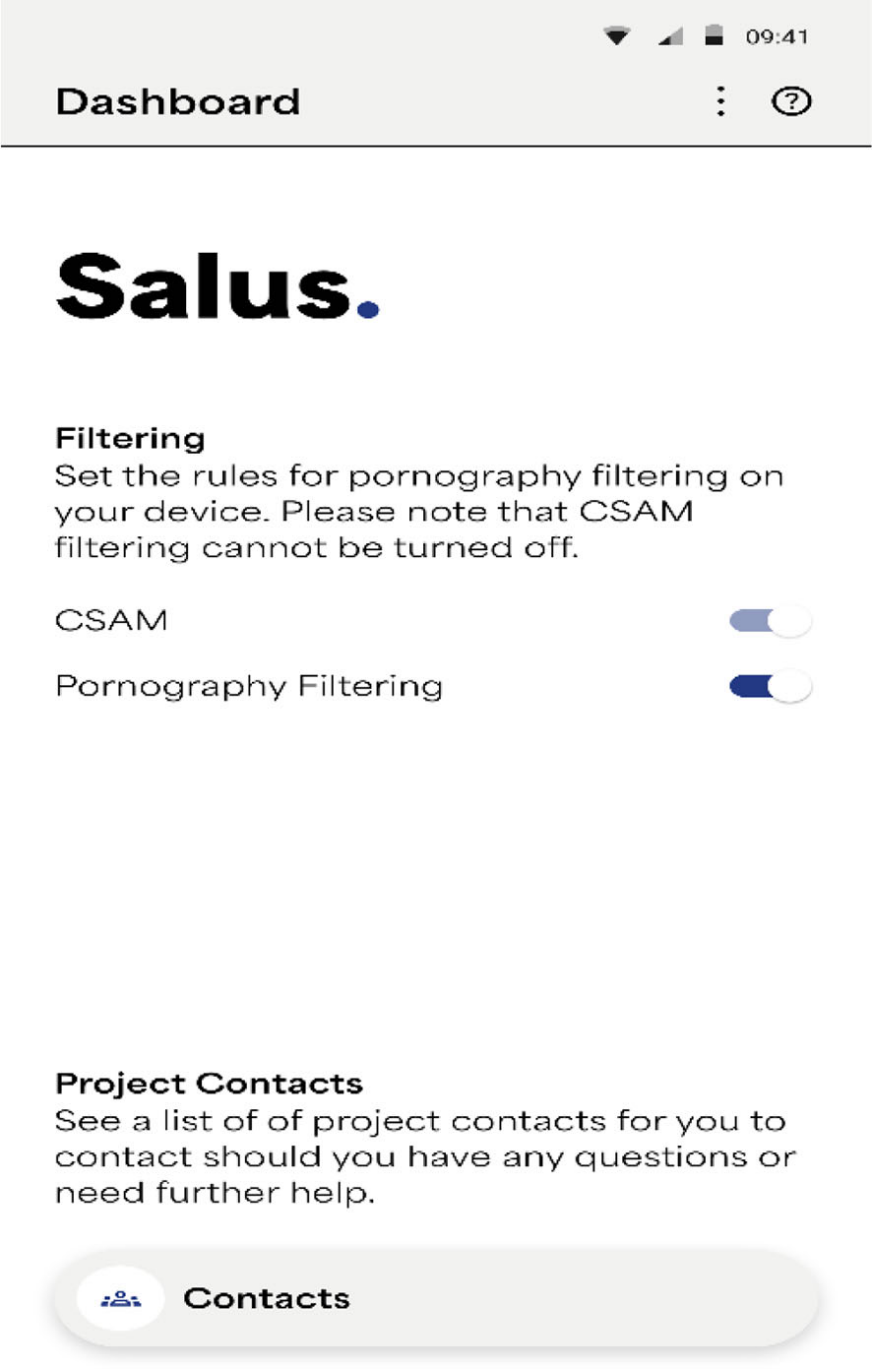
Salus prototype optional pornography filter.

Most interviewees and FGD participants who reported that Salus should block adult sexual content also commented that app users should have the option to switch such a pornography filter on and off, see **Figure 3**.

It should be highlighted, however, that not all interviewees reported that Salus should also block adult content. Some interviewees (n = 3) reported concern that blocking all pornographic materials would lead them to lose a ‘healthy outlet’ for their sexual preferences – which they regard as being provided by pornographic content depicting young, but adult, performers.

Interview and FGD participants also discussed their thoughts regarding what other sites and pages Salus should block. Some participants (four interviewees; participants from three FGDs) suggested that Salus should block certain social media and gaming sites, such as TikTok, Facebook, Facebook Messenger, Snapchat, and Discord. By blocking these sites, Salus would remove the ‘temptation’ for Salus users to access images and videos of children (which may be a pathway to viewing CSAM). Blocking these sites would also remove the users’ ability to attempt communication (chats) with children.

*“Discord, Snapchat … Make it no longer accessible.” – Interview participant 18 (Belgium).*

Additionally, 10 interviewees and participants from three FGDs recommended that Salus should also block users’ access to the dark web, as blocking the dark web would significantly reduce offenders’ ability to access CSAM and engage with other CSAM offenders. However, there was no clear consensus regarding whether dark web blocking should be optional or mandatory.

Finally, 11 interview participants and participants from two FGDs recommended that Salus should block not only images and videos, but also audio clips, websites, pop-up ads, and written CSAM, as for some individuals, these materials may provide a pathway to viewing CSAM:

*“For someone like us, we have to be very cautious [ … ] because indeed, we know that clicking through to [CSAM] often happens in this way, [ … ] they actually advertise on other websites.” - Interview participant 21 (Belgium).*

#### CSAM block alerts

There was consensus in the interviews and FGDs that there should be a CSAM block alert when an individual attempts to access CSAM, but that the alert should not be loud or obtrusive:

*“In relation to privacy [ … ], if you make it the world’s most unique sound then please don’t do that.” – Interview participant 15 (Belgium).*

**Figure 4:**
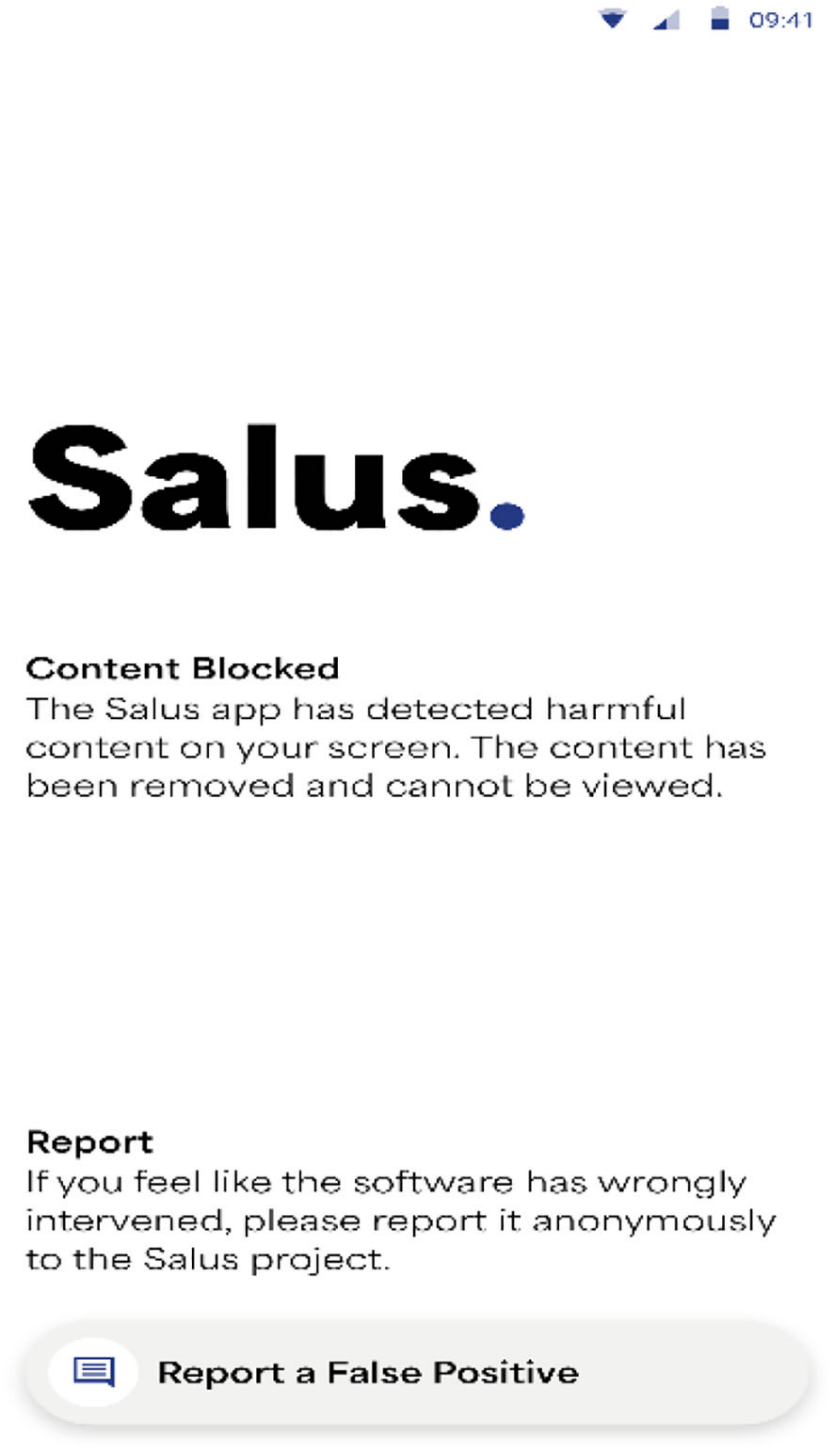
Salus prototype content block message.

There was considerable variation in participants’ suggestions regarding block messages. Eleven interviewees and participants from one FGD suggested that a CSAM block message should be neutral, along the lines of ‘content blocked’, see **Figure 4**.

Others (12 interviewees; participants from two FGDs) suggested that the block messages should be helpful and direct users to available support. Still others (four interviewees, participants from one FGD) suggested that block messages should be personalized, that is, that the user could draft their own block message that may serve as a personal deterrent to accessing CSAM. For example, “if you don’t stop, you’ll lose your family.” Three interviewees and participants from three FGDs further suggested that users should have the option to change block messages at their convenience, so that they do not become ‘numb’ to any one message as a result of seeing it too frequently.

Our findings thus highlight the principle of adaptive notification systems. Deterrence notifications should remain effective over time, while avoiding stigmatizing or attention-drawing alerts. Participants highlighted that they have a preference for subtle or customizable notifications (“if you make it the world’s most unique sound then please don’t do that”), thus, app designers should implement subtle, customizable blocking notifications that maintain deterrent effectiveness without causing desensitization. This may include user-selectable notification styles, message personalization options, and message rotation capabilities.

### Interactivity

Interview and FGD participants were also asked to discuss their thoughts on Salus interactivity options, that is, what features could be included in the prototype that would enable users to interact with the app, and the app developers. Interview and FGD participants suggested that the following features may be useful: A diary function; a personal CSAM statistics page; a support resources section; a Salus FAQ section; and a function to allow users to provide anonymous feedback on prototype functionality to the app developers.

Interviewees suggested that the diary function would be helpful for users to record and reflect on their engagement with Salus. Interviewees explained that having a diary function could be useful for analysing the user’s personal behaviour, that is, their attempts to access CSAM; this might help them prepare prior to therapy sessions. Some interviewees further suggested that the diary function would be useful for noting and reflecting on how many times they attempted to access CSAM. Interviewee and participants in two FGDs suggested that, in addition to a function for writing one’s thoughts, that the app could allow users to use emojis to express themselves. That is, that the user could record, on a particular date, a smiley face to reflect that they have had a positive day in terms of interaction with Salus, or a sad face to reflect that they attempted to access CSAM that day.

The interview sample was divided nearly evenly in terms of interviewees who reported that a diary option would be beneficial and those that did not. Fifteen interview participants suggested that they would like to see a diary function in the Salus prototype, while 13 others reported that they would not use the diary function; however, the latter group reported that they would not mind having the function in the app, even if they were not interested in using it.

*“For me, it (a diary function) wouldn’t work. But I understand the need for it.” – Interview participant 13 (Belgium).*

Nearly all study participants (22 interviewees and three FGDs) said that it would be beneficial for Salus users to have a personal statistics page within the app where they could see how many times Salus had blocked CSAM during a specific period, that is, during a week or month. They were in favour of this function because it could act as a motivating factor for preventing CSAM viewing:

*“If you use the app and can see your statistics you’ll think ‘whoa, last month I had a pop-up twenty times, and this month I only had it five times’, which gives you a positive affirmation of ‘hey. Well done. I’m doing well, I’m well on my way to recovery.’” – Interview participant 12 (Belgium).*

Four interviewees further suggested that users could share these statistics with their service provider, for discussing their progress with regards to reduced CSAM viewing.

Seven interviewees further suggested that positive messaging when users open the personal statistics page could be beneficial to motivating them to cease viewing CSAM. For example, messaging along the lines of:

*“Well done! You have not tried to access and blocked content for X days.” – Interview participant 4 (United Kingdom).*

However, two interviewees and participants from two FGDs did emphasize that, for those who experienced an increase in a week or month in terms of CSAM blocks, the statistics page could have a negative effect on an individual’s motivation to stop viewing CSAM, therefore the personal statistics page should be optional for users, and users should be able to switch the personal statistics page on and off as they please.

Both interview (n = 26) and FGD (n = 3) participants suggested that a dedicated resources page within Salus would be helpful for users, particularly those who have recently entered a support service and are beginning therapy:

*“I believe it could be [a valuable addition to the app], as many people may not take that step or even be aware of available resources.” – Interview participant 13 (Belgium).*

They suggested that such a resources page could include a list of approved helplines and service providers in their country; links to self-help resources (e.g., books, therapy modules etc); and more general mental health resources. Interviewees and FGD participants highlighted that such resources might be particularly helpful for app users who are at the beginning of therapy, or have recently been convicted of a CSAM offence.

Finally, both interview (n = 22) and FGD (n = 1) participants suggested that there should be a function within Salus to provide technical feedback to the app developers. Interviewees highlighted that it would be relevant for users to be able to anonymously report false-negative CSAM blocks, meaning that the app blocked something as CSAM when the user did not attempt to access CSAM, as well as failures of the app to block CSAM and/or adult content. This kind of reporting to app developers would enable constant refinement of the app during the pilot.

These findings highlight the app design principle of optional interactivity with user control. While participants highlighted the value of app optional features, such as CSAM statistics tracking and diary functions (“I believe it could be [a valuable addition to the app]”), they also identified the potential for unintended negative consequences associated with such features. Thus, app designers should design interactive features as opt-in components with granular permission controls, allowing users to enable/disable features independently.

### Deployment

The area of questioning that most study participants struggled with in terms of sharing their thoughts, opinions, and concerns was that of app deployment. Many interview and FGD participants admitted that they had limited understanding of how apps such as Salus can reasonably be deployed, ensuring the privacy and anonymity of users.

Some interview (n = 13) and FGD (n = 2) participants suggested that Salus should only be accessed through a secure link that is provided to app users by the service provider. They further suggested that, at the same time, or even prior to sending the download link, users are sent the FAQs and download instructions so that they have a full understanding of how to download Salus, and common problems in app download and Salus usage. This would ensure that users feel ‘safe’ in the sense that the download link and FAQs have been provided by their service provider, and the user commences the Salus download with a full understanding of how Salus works.

Other interview participants (n = 6) suggested that the app could be downloaded through an online store, for example the App Store or Play Store, but they emphasized that there are security risks and concerns associated with downloading Salus from online stores, and that users would require written reassurance that the app is ‘safe’, and that Apple and other companies will not store or share their data.

Finally, there was consensus in the interviews and FGDs that the download should not require any personal details, such as a user’s name or email address, highlighting the importance of privacy and anonymity at the point of app download.

These findings highlight the key app design principle of trusted-channel deployment. Participants highlighted comfort with apps distributed through trusted service provider channels, as such deployment would reduce their concerns about app legitimacy and any data collection by the app developer. App designers and researchers should thus prioritize app deployment, where possible, through established therapeutic relationships (that is, through service providers), rather than public channels. This can be achieved through secure app distribution links via service providers, and supported through comprehensive pre-deployment information, such as the FAQs and other activities highlighted in earlier paragraphs.

### Benefits and concerns

Interview and FGD participants were also asked their thoughts on the potential benefits of Salus for users, as well as any concerns that had not already been raised during the interview or FGD. There was consensus across the interviews and FGDs that Salus could have immense benefits to users, particularly when combined with therapeutic support from their service provider. However, it should be emphasized that there was likely interview sample bias, as participants were recruited through service providers and were actively seeking help to stop CSAM viewing. Thus, their perspectives are undoubtedly skewed in favour of technological solutions to prevent CSAM consumption.

Some interviewees expressed concern that Salus may constantly block websites and content that are not CSAM related, or interfere with internet connectivity, thus negatively affecting users’ ability to work or use the internet for other purposes:

*“If it keeps blocking things that are not at all dangerous and I want to use my mobile phone normally again, then I would uninstall this app.” – Interview participant 2 (Germany).*

However, the same interviewees emphasized that they would tolerate a certain threshold of internet interference and incorrect blocking in light of the potential therapeutic benefits of Salus:

*“It would be a bit worse for user-friendliness if you were surfing the internet and suddenly it said: Website unavailable. There can be many reasons for this. And then I would first have to log into the app and see if it was blocked. If this were to happen frequently, it would be extra work. But I would be prepared to put up with it if the benefit is there for me.” – Interview participant 2 (Germany).*

Finally, some interviewees and FGD participants raised concerns that users may become reliant on the app. These participants were essentially worried that, at the end of the pilot period, users might experience distress or a relapse because Salus was no longer available to them, and they did not have a substitute tool to prevent CSAM viewing, highlighting the importance of Salus users also receiving regular therapeutic and other support:

*“It would be important to continue that regular support … to avoid any relapses.” – FGD participant (United Kingdom).*

These findings highlight the principle of fail-safe harm prevention. Participants identified the potential risks for app users associated with sudden app unavailability, highlighting the need for supported transitions. Projects should be designed for supported app exit, ensuring that there is no abrupt withdrawal, which could lead to unintended negative consequences for app users. This can be achieved through phased deactivation protocols, alternative resource provision, and continuity planning.

## Discussion

A key strength of this study is that Salus is a novel and much needed tool, which offers substantial scope for scalability and impact in a highly challenging ethical and legal context, within the EU, and indeed, globally. As noted in the Introduction of this paper, a high number of at-risk individuals want help – including technological interventions – to stop viewing CSAM. The ReDirection survey (2021) found that only 13 per cent of survey respondents had sought help but that 50 per cent reported that they would like to stop viewing CSAM, and 62 per cent had tried to stop viewing CSAM but failed (14). Thus, Salus and similar apps, if effective at blocking CSAM, may have significant practical and therapeutic benefits for individuals who want to stop viewing CSAM.

Another strength of the study is the UCD approach adopted, whereby the voices of at-risk individuals, and service providers are the centre of app design. A UCD approach to the design and development of Salus and similar apps is the best approach for ensuring the highest chance of success in terms of app usability, effectiveness and positive outcomes for users. A related strength is how the UCD approach to the design of Salus was the first step in the project, prior to the further testing and refinement, and piloting and evaluation of the app. The literature highlights the importance of developing apps such as Salus over several phases or stages. The first is the UCD of the app, inviting potential users to provide their insights regarding the app design, functionality, and deployment. The second is to test and strengthen the app. The third is to pilot and evaluate the app. In a recent study regarding the design and usability evaluation of a mobile application for self-care among Iranian adolescents, for example, the research and evaluation team first gathered the insights of potential users through semi-structured interviews, before applying the interview findings to the app development, and, finally, evaluating the app effectiveness through piloting of the app for two weeks and gathering feedback through the MAUQ questionnaire (31). Similarly, Para et al. (2024) (32) discuss how they designed and evaluated a web-based app for parents to reduce child abuse. Para et al. (2024) (32) discuss how they developed the app over three stages – first, inviting potential users to share their thoughts and opinions on app functionality; then applying the results to the app design; and finally, evaluating the app through a piloting phase. Thus, the literature highlights the importance of UCD in app development, as well as subsequently conducting extensive piloting and evaluation of such apps over a period of time, and rigorously evaluating the results of pilots before the app is further developed and deployed to a wider population. As noted in previous sections of the paper, this current paper presents the findings from the UCD and development of Salus, while future papers will present the findings from the pilot and evaluation of the app.

A final strength is that the interviews with at-risk individuals were conducted by their service providers. In Belgium and Germany, where most interviewees were recruited from the pool of existing service users, this meant that the interviews with at-risk individuals were conducted by someone that they already know and trust. As highlighted by Dugas et al. (33) such trust is important when the interviewees are vulnerable individuals and may be wary of researchers and how their interview data may be used. As further highlighted by Dugas et al. (33) when interviews are conducted by service providers there is a better chance of success in participant recruitment, and increased likelihood of power imbalances being reduced. Thus a key insight for organizations developing similar apps is that, where possible, participants should be recruited via organizations that are providing support to individuals, and the same service providers should conduct data collection. It is further important to highlight at the point of informed consent that declining to participate in the project, or later withdrawing from the project, will in no way affect individuals’ access to services offered by the service providers. For the current project, we included such a statement in the information sheet for participants. These measures will ensure a better response to interview and other data collection requests, as well as reassure participants that the project and the wider study can be trusted, and that participation in the project will in no way affect access to normal service provision.

In terms of important findings from the study, privacy and security emerged as paramount concerns for individuals at self-reported risk of viewing CSAM and were highlighted by FGD participants as features requiring critical attention during app design and development. Interviewees and FGD participants expressed concern about the potential discovery of the app by family members, friends, or colleagues, which could inadvertently expose their behaviour related to CSAM. These concerns are consistent with previous research on digital mental health interventions, which have shown that privacy is a significant barrier to adopting and sustaining such tools (34). In the context of at-risk users, the fear of discovery is particularly acute, given the illegality and stigma surrounding CSAM consumption, which could result in negative consequences, such as social exclusion or legal repercussions.

The issue of app discovery underscores the need for app design features that prioritize anonymity and discretion, a finding that aligns with the results of other studies on sensitive health applications for marginalized groups (see, for example, 35). The participants in our study – both interviewees and FGD participants - proposed that Salus should adopt a neutral and inconspicuous design to prevent accidental discovery by others. Suggestions included using an abbreviated app name or using an app name that provides no insights into what the app is for. These recommendations are consistent with the principles of ‘privacy by design,’ which advocate for minimizing the likelihood of exposure to third parties while maximizing user control over their data (36).

Furthermore, concerns regarding data security and the risk of hacking were prevalent among participants. The unwanted use of personal data by third parties is a common concern regarding using mobile devices for health-related activities. This is an essential consideration for all digital interventions, but it is particularly salient for at-risk populations, where data breaches could lead to blackmail or criminal exposure, and little support for dealing with data breaches is available (37). This reflects the growing recognition of the need for robust technical safeguards to ensure the confidentiality and integrity of users’ personal information in digital health platforms.

Finally, interview and FGD participants raised concerns about potential legal consequences, fearing that law enforcement might access information related to Salus usage. This concern echoes discussions about digital interventions to reduce other types of offending such as illicit drug use (38). To mitigate these concerns about potential for unintended legal exposure, participants in our study suggested that Salus should include a clear privacy statement emphasizing that the application is not affiliated with law enforcement agencies and that there are no legal consequences for using the app.

Participants (interviewees and FGD participants) endorsed the inclusion of optional adult sexual content blocking functionality within the application. Many recognized the critical role that such a feature could play in preventing the consumption of illegal CSAM, as adult sexual content is often a pathway to viewing CSAM (39). Blocking functionality has been widely advocated in the literature as a tool for behavioural change, particularly in addiction-related interventions, such as for pornography reduction (40), but evidence of efficacy is scarce.

Interview and FGD participants also expressed concerns about the potential effectiveness of block mechanisms, especially in light of the adaptive nature of online environments. Some participants expressed concern about the stigmatizing effect of a visible block alert. This points to the need for subtle and quiet block notifications. Study findings also pointed to the need for app users to be able to personalize their CSAM deterrence message, so that these messages continue to serve as effective deterrents for the individual.

Regarding interactivity, interview participants were generally open to features allowing for personalized engagement within the application, such as a diary or personal statistics stage for self-reflection on the app experience. Research shows that such interactivity features can elicit app engagement (41). For users seeking help for CSAM-related behaviours, interactive features could serve as a source of support and motivation, providing users with a sense of agency and control over their recovery process.

Interview and FGD participants also suggested that the app should offer additional resources or links to professional support. This is consistent with findings from digital mental health interventions, highlighting the importance of integrating professional care options into apps to enhance their therapeutic potential (42). Given the sensitive nature of CSAM-related behaviours, incorporating interactive features that offer users actionable steps and immediate feedback could be crucial in helping them feel empowered to change their behaviour. Promising, but not yet robust effects have been shown for information and communication technologies, such as on-device apps with cognitive-behavioral elements, at reducing addictive behaviours (43), and personalized mobile interventions are promising in moderating lifestyle behaviours (44).

A key finding from our study was that while interactive features should be included in the app design, users should be able to choose which, if any, interactivity features they want to engage with. Furthermore, users should be able to switch on and off interactivity functions as they please. This is critical for ensuring that any unintended effects from interactive features – such as a mental health decline due to notifications that their CSAM consumption has increased – are able to be quickly managed.

The study participants emphasized the importance of deployment methods that ensure accessibility and security and privacy, particularly for individuals reluctant to seek help through traditional channels. Research conducted by Hayes et al. (45) highlighted concerns regarding the collection and sharing of personal data conducted by mobile apps without the knowledge or consent of the user. It is critical that at-risk individuals who volunteer to use apps such as Salus have a full understanding of how the app is to be securely downloaded onto their device/s, the security and privacy features of the app, and what data will be collected, as well as what data will not be collected. A key learning from our study is that, where possible, CSAM blocking app deployment should be done via a secure link that is provided by a service provider.

Beyond the four critical focus areas, interview and FGD participants also shared their overall impressions of Salus and their concerns regarding its potential to help users. Many participants believed the application could provide a valuable resource for individuals struggling with CSAM consumption, offering an anonymous and private space for users to engage with the app. However, as noted in previous sections of the paper, the interviewees in this study were adults wanting to stop their CSAM consumption, thus their views on the value of Salus and similar technology are no doubt skewed in favour of the app. Interview and FGD participants also shared concerns regarding users’ abrupt withdrawal from the app, and suggested that this should be considered by app developers and service providers with a view to ‘phasing’ users out of the app while providing access to alternative technological solutions, advice and therapy. Thus, a key finding of the study is that, for projects with set timelines, app users should be phased out of app usage over time, and also offered alternate technologies to reduce their CSAM consumption (or other harms). It is critical that app usage is not suddenly withdrawn as this may lead to reversals in any mental health gains as a result of loss of app usage.

Based on the empirical findings from the UCD of Salus, we propose a framework of design principles that may guide the development of on-device harm-reduction technologies. These principles synthesize participant insights into actionable guidance for app developers and researchers working on similar interventions. **Table 2** summarizes the principles, linking them to the major themes identified (concerns shared by participants), and the related design implications.

**Table 2:** Principles, themes, and design implications.

Principle	Themes (participant concerns)	Design implications
Privacy by default architecture	Significant concerns regarding data security and privacy. Concerns regarding legal consequences of app usage (e.g., background reporting of the app to law enforcement).	Privacy protections should be embedded at system level. App should adopt a discreet design: - App icon should be discreet and neutral. - Full app name should not be used. App should not require regular log in (one-off initial log in). App should minimize data collection to the extent possible (not track user data, such as location). Encryption and anonymization by default. Only minimal data should be passed to the server, and this should be done in encrypted and anonymized format. Server should be based in a secure location.
Discretion through design ambiguity	Concerns regarding potential discovery of app usage by users’ family members, colleagues, and/or friends.	App designers should create deliberately ambiguous app visual and naming conventions that prevent app discovery: - Neutral iconography. - Abbreviated naming. - Quiet background operation after initial setup.
Adaptive notification systems	Deterrence message effectiveness may wane over time. Loud notifications may attract unwanted attention.	Deterrence messages should be adaptable, allowing users to set, and frequently change, their own deterrence messages. Notifications should be quiet and subtle.
Optional interactivity with user control	Compulsory interactivity features may be detrimental to users’ mental health.	App should build in: - Optional pornography filter, which can be switched on/off at user’s discretion. - Optional additional content blocker (e.g. social media sites). - Personal CSAM statistics page. - Diary function. - Self-help resources. - Anonymous feedback to developer.
Trusted-channel deployment	App deployment through public channels may not be trusted.	Where possible, app should be deployed through a link disseminated by a service provider (trusted organisation). Download should not require any personal details.
Progressive trust building	Lack of up-front information about the app may reduce trust and take-up.	App developers and research teams should develop transparency mechanisms that proactively address user concerns: - A privacy statement that outlines what data will be collected, how it will be used etc). Statement should make clear that there are no legal consequences from app usage. Statement should further highlight that app is not connected in any way to law enforcement. - Frequently Asked Questions document.
Fail-safe harm prevention	Abrupt cut-off of app access may have negative consequences for users’ mental health.	Project teams should ensure that: - There is no abrupt withdrawal from app usage. - Users have access to phased deactivation protocols, alternative resource provision (i.e. access to an alternative app), and continuity planning.

Principle 1: Privacy-by-default architecture. Our findings demonstrate that privacy concerns (discovery of the app, data security, legal exposure) constitute primary barriers to app adoption among at-risk populations. App designers should embed privacy protections at the system level. This principle requires that no data is collected on the participants’ use of the app, app usage is protected through end-to-end encryption, and the app operates locally on-device where possible.

Principle 2: Discretion through design ambiguity. Our study identified that fear of social exposure is a major concern for potential app users, requiring design solutions that protect app users from unintended discovery. App designers should create deliberately ambiguous visual and naming conventions that prevent inadvertent disclosure, such as neutral iconography, abbreviated naming, and quiet background operation after initial setup.

Principle 3: Adaptive notification systems. Participants emphasized the need for notifications that remain effective over time while avoiding stigmatizing or attention-drawing alerts. App designers should implement subtle, customizable blocking notifications that maintain deterrent effectiveness without causing desensitization, such as user-selectable notification styles, message personalization options, and rotation capabilities.

Principle 4: Optional interactivity with user control. While participants valued optional features, such as CSAM statistics tracking and diary functions, they also identified the potential for unintended negative consequences, necessitating user control. Thus, app designers should design interactive features as opt-in components with granular permission controls, allowing users to enable/disable features independently.

Principle 5: Trusted-channel deployment. Participants expressed greater comfort with apps distributed through trusted service provider channels, reducing concerns about app legitimacy and data collection. App designers and researchers should prioritize app deployment through established therapeutic relationships rather than public channels. This can be achieved through secure app distribution links via service providers, and comprehensive pre-deployment information.

Principle 6: Progressive trust building. Trust emerged as foundational to app adoption, requiring explicit communication strategies that address users’ specific fears. App developers should design information architecture that builds user confidence through transparency. This should include clear, accessible privacy statements, FAQ sections, and evidence of independence from law enforcement.

Principle 7: Fail-safe harm prevention. Participants identified risks associated with sudden app unavailability, suggesting the need for supported transitions. Projects should be designed for supported exit rather than abrupt withdrawal. This can be achieved through phased deactivation protocols, alternative resource provision, and continuity planning.

Our principles are situated with the existing privacy-by design frameworks, which discuss concepts such as early user engagement and iterative co-design (46). Our principles contribute to the existing frameworks by transferring the principles into actionable, context-specific design imperatives for populations facing criminalization, stigma, and legal exposure. For example, ‘privacy-by-default architecture’ is common in privacy-by-design literature (47) but our principle specifically addresses the unique needs of at-risk individuals who fear both social discovery and law enforcement intervention. The novelty of the seven principles that we highlight in this paper is that - they are more specific than generic UCD or privacy-by-design principles; they are more generalizable than app-specific design guidelines; and they provide theoretical grounding for practical design decisions.

There were four limitations to the study, which should be highlighted. The first limitation relates to the interview sample. The interviewees were at-risk individuals who are currently being supported by a service provider to stop viewing CSAM. They have sought help from the service provider because they want to stop viewing CSAM. Thus, the thoughts and views of the interviewees on, for example, the value of the app are undoubtedly skewed. Had we recruited more broadly (outside the service providers), we would no doubt have gathered data that would suggest that, at least for some at-risk individuals, such a tool is not wanted or needed.

A second limitation of the study is the sample size. While 31 interviews was an adequate number of interviews in other to gather sufficient data to answer the research questions, a higher number of interviews may have resulted in different or additional insights into what Salus should look like, its functionality, and deployment methods, and thus different or additional design principles. Similarly, additional FGDs could have resulted in different findings with regards to Salus design, functionality, and deployment, and also different design principles.

The third limitation is two-fold: We approached the interviews with four established domains for questioning, and we collected data from interviewees only once. For this project, with its strict two-year timeline for completion, the four domains were necessary in order to collect the information that was needed by the app developer to design the prototype. The timeline also meant that we only had time to approach interviewees once about their thoughts and concerns regarding app design. Our approach thus reduced inductive flexibility. Thus a key learning for project teams developing similar apps is that, where possible, teams developing similar technology should ensure that the project allows adequate time for iterative design. Potential app users should be invited to contribute their thoughts and opinions on prototype design, then, later, their opinions on app functionality after they have trialled the app for a period of time. In this way, app development will be truly couched in a UCD framework and follow an iterative process.

The fourth limitation was that interview results cannot be considered quantitative evidence. Not all the proforma questions were asked by those conducting the interviews (or the prompts provided in the proforma). For example, when reporting that a certain number of participants said that a diary function would be helpful, other interviewees and FGD participants may have reported the same, but were not asked the prompt question by the interviewer/FGD coordinator. Thus, reported numerical indicators in this paper can serve only as illustrative measures of consensus, rather than definitive quantitative evidence.

## Conclusion

By synthesizing participant insights into seven explicit design principles, we provide a conceptual foundation for developing harm-reduction technologies that balance effectiveness with user autonomy, safety with accessibility, and behaviour change with privacy protection. These principles - privacy-by-default architecture, discretion through design ambiguity, adaptive notification systems, optional interactivity with user control, trusted-channel deployment, progressive trust building, and fail-safe harm prevention - represent design imperatives derived from analysis of user needs and concerns. They advance the field both methodologically, by demonstrating how UCD research can contribute to design science, and practically, by providing actionable guidance for developers and researchers working across various harm-reduction contexts.

Future research should test these principles across different technological interventions and populations, refining them through iterative application and evaluation. Longitudinal studies examining how these principles influence tech adoption, sustained use, and behavioural outcomes would further validate and enhance the principles and framework.

We conclude this paper with a reflection that the ultimate goal is not merely to design better apps that prevent CSAM viewing, but to contribute to a culture of prevention that effectively reduces harm while respecting the dignity and autonomy of at-risk individuals. Technological interventions such as Salus represent one component of a comprehensive public health approach that must include accessible therapeutic services, evidence-based treatment options, and societal efforts to reduce stigma and facilitate help-seeking. Our principles provide a foundation for developing technologies that can complement these broader efforts, offering scalable, accessible, and effective tools for individuals struggling with harmful behaviours.

## Data Availability

The datasets presented in this article are not readily available because Dataset is interviews with adults at self-diagnosed risk of viewing child sexual abuse material. Requests to access the datasets should be directed to deanna.davy@aru.ac.uk.

## Footnotes

1 The Protech project leverages multi-disciplinary expertise across four EU therapeutic providers, two academic institutions, one NGO, and one technology company to develop, implement and evaluate the effectiveness of a user-centered, on-device, safety tech prevention tool to prevent the viewing and distribution of CSAM for both pre- and post-offending individuals.

2 British pound sterling.
